# Family Caregivers’ Provision of Teeth Care to Older Adults: The Role of Caregiver Stress, Care Demands, and Training

**DOI:** 10.1093/geroni/igaf122.3770

**Published:** 2025-12-31

**Authors:** Yuanchang Zhao, Mengzhao Yan

**Affiliations:** Case Western Reserve University, Cleveland, Ohio, United States; University of Southern California, Los Angeles, California, United States

## Abstract

Teeth care is essential not only for older adults’ dental health but also for their overall quality of life. However, family caregivers often overlook this aspect of care. Limited studies have identified the factors influencing caregivers’ actions in providing teeth care, especially using nationally representative data. Guided by the caregiving reluctance framework and the caregiver stress model, this study aims to examine factors associated with family caregivers’ provision of teeth care. Using Round 13 of the National Study of Caregiving (NSOC) data (N = 2,557), we employed nested logistic regressions to test the effects of care demands, caregiving training, and stressors on the provision of teeth care, controlling for demographic characteristics. We found that stressor variables (longer caregiving hours, OR = 1.1; caring for someone with a disability, OR = 2.6; caregiver overload, OR = 1.18), demand variables (dementia, OR = 2.58; chewing problems, OR = 1.65), and training (OR = 1.92) were all linked to higher odds of providing teeth care. We also found that caregiver stressors, demands, and training variables were positively correlated. These findings suggest that caregiving stressors, demands, and training are intertwined in influencing the provision of teeth care. Furthermore, the association between caregiving stress and teeth care provision signals potential mental health risks associated with providing such care. This study underscores the importance of training family caregivers to recognize and provide effective teeth care for older adults and offers insights for public policies aimed at improving older adults’ dental health and supporting caregivers.

